# Total Ankle Arthroplasty: A Comparative Review of Surgical Approaches and Outcomes

**DOI:** 10.2106/JBJS.OA.26.00019

**Published:** 2026-06-15

**Authors:** Benjamin Hershfeld, Pranav Krish, Pedro Smith, Anthony Modica, Alexandra Krez, Brandon Klein, Adam D. Bitterman

**Affiliations:** 1 Northwell Health, New Hyde Park, New York, New York; 2 Department of Orthopaedic Surgery, Northwell Health—Huntington Hospital, Huntington, New York; 3 Donald and Barbara Zucker School of Medicine at Hofstra/Northwell, Hempstead, New York; 4 Department of Orthopaedic Surgery, Northwell Health—Long Island Jewish Medical Center, Queens, New York; 5 Department of Orthopaedic Surgery, University of Pennsylvania Perelman School of Medicine, Philadelphia, Pennsylvania

## Abstract

» Total ankle arthroplasty (TAA) has evolved from an implant-limited procedure to a technique-sensitive operation, with surgical approach selection emerging as a key determinant of soft-tissue complications, deformity correction, and alignment accuracy.

» Anterior and lateral transfibular approaches offer distinct advantages and tradeoffs in visualization, angiosome preservation, deformity correction, and technical complexity, and should not be viewed as interchangeable corridors.

» Anterior approaches remain the most widely used and compatible with most implant systems but carry the highest risk of wound complications, particularly in patients with compromised anterior soft tissues or vascular disease.

» The lateral transfibular approach provides extensile exposure and facilitates coronal-plane deformity correction while preserving anterior skin perfusion, at the cost of added bony morbidity, increased technical demands related to fibular osteotomy and fixation, and constrained revision options because failed lateral systems often require revision through an anterior approach using alternative implants.

» Optimal outcomes in TAA depend less on the chosen approach itself than on appropriate patient selection, meticulous soft-tissue management, implant-approach compatibility, and surgeon experience, emphasizing the need for individualized, strategy-driven approach selection.

## Introduction

End-stage ankle arthritis is a highly debilitating condition, affecting approximately 1,000 per 100,000 individuals^1^. Unlike hip and knee osteoarthritis, which is predominantly idiopathic, over 90% of ankle arthritis is post-traumatic^2,3^. When conservative treatment fails, surgical management becomes necessary, with arthrodesis and total ankle arthroplasty (TAA) representing the principal options.

Arthrodesis has traditionally been the gold standard, offering durable pain relief but sacrificing motion and normal gait mechanics^4,5^. Modern TAA aims to preserve mobility and physiologic biomechanics while reducing adjacent joint stress^6,7^. Although early implants had poor outcomes, third-generation systems and refined techniques now provide pain relief comparable with fusion, with superior gait restoration but a modestly higher midterm revision risk^8-12^. Patients with advanced ankle arthritis are often younger and more active than typical hip or knee arthroplasty patients^1,13-15^. Importantly, “younger” is not uniformly defined in the TAA literature and should be interpreted relative to the traditional older, lower-demand TAA population rather than as a strict age threshold.

Traditionally, TAA was reserved for older, low-demand patients with minimal deformity because these factors were associated with lower complication and revision rates. Although indications have expanded with improvements in implant design and surgical technique, evidence in younger patients remains mixed. Accordingly, TAA in younger patients should be approached cautiously, with nuanced discussion of expected functional benefit, revision risk, and the potential need for future reoperation^16^.

## Historical Evolution of TAA

Surgical treatment of end-stage ankle arthritis has evolved substantially. Early TAA designs introduced in the 1970s were highly constrained and cemented, and high rates of loosening, subsidence, and early failure led to widespread abandonment of the procedure^17-19^. During this era, failure was predominantly implant-driven, reflecting limited understanding of ankle biomechanics and fixation requirements, and surgical exposure was viewed as a secondary technical consideration rather than a determinant of outcome^19^. Later generations improved fixation, reduced constraint, and better reproduced ankle anatomy, allowing TAA to re-emerge as a viable motion-preserving alternative to arthrodesis^17^.

As implant-related failure declined, the importance of soft-tissue management, deformity correction, and component alignment became more apparent. As indications expanded beyond the traditional older, low-demand patient population to include greater coronal and sagittal deformity and more complex primary and revision cases, surgical approach selection became increasingly consequential^4^. Anterior and lateral transfibular approaches evolved in response to the limitations of earlier techniques. Laterally inserted trabecular metal systems introduced a distinct porous ingrowth technology and now have substantial mid-term follow-up, but contemporary anterior systems have also evolved considerably, making it increasingly difficult to attribute differences in outcomes to approach alone^20,21^.

Once TAA is selected, determining the surgical approach becomes a critical part of operative planning. Unlike hip and knee arthroplasty, approach in TAA is closely linked to implant design and instrumentation. Most contemporary systems are designed for anterior insertion, whereas lateral TAA is limited to specific side-entry platforms^22^. Accordingly, comparisons between anterior and lateral TAA should not be interpreted as comparisons of exposure alone, because reported differences may also reflect implant-specific features, including fixation strategy, bearing technology, and revision options.

## Surgical Approaches and Exposure

Modern TAA relies primarily on anterior and lateral transfibular exposures. Approach selection is a strategic decision shaped by implant access, soft tissue considerations, deformity correction, and revision implications. Within these categories, multiple incision variants, accessory windows, and fixation strategies may be used depending on the soft-tissue envelope, deformity, and concomitant procedures. Anterior-based exposures remain the most common and compatible with most implant systems, whereas the lateral approach may be advantageous in selected deformity or soft-tissue scenarios.

### Anterior Approach

**Fig. 1:**
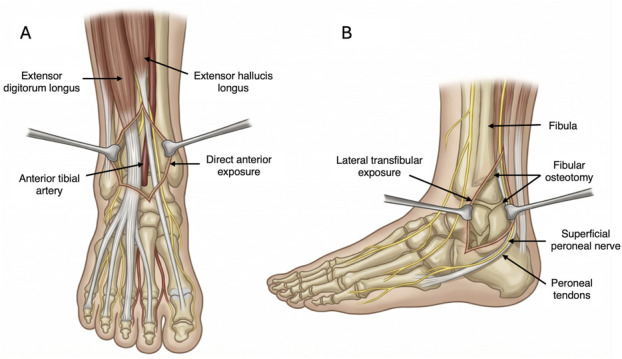
Operative exposure anatomy for anterior and lateral transfibular TAA. **Fig. 1-A** The anterior exposure to the ankle joint, highlighting the extensor digitorum longus, EHL, anterior tibial artery, and the direct anterior exposure interval. **Fig. 1-B** The lateral transfibular exposure, highlighting the fibula, fibular osteotomy, superficial peroneal nerve, peroneal tendons, and the lateral transfibular exposure corridor. EHL = extensor hallucis longus, TAA = total ankle arthroplasty.

**Fig. 2:**
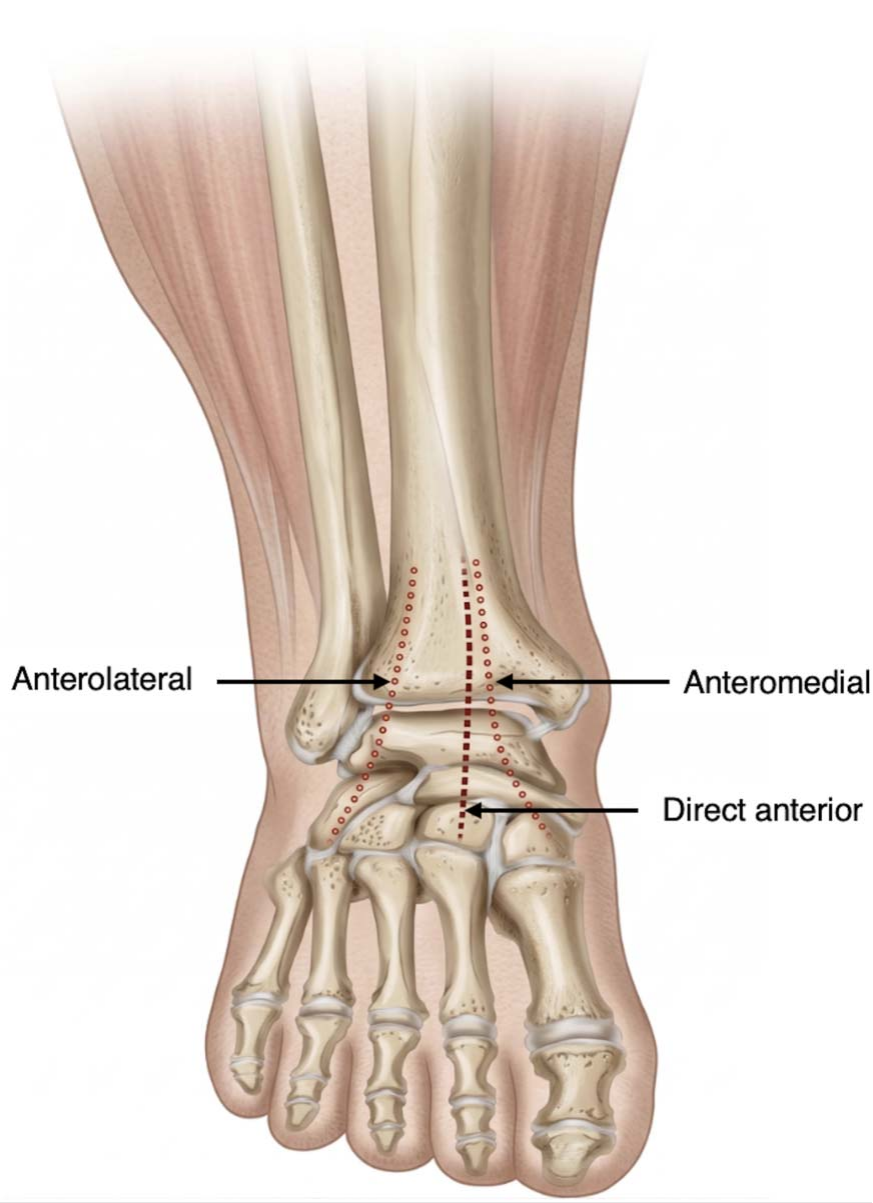
Anterior surface incision options for TAA Surface incision variants for anterior TAA are shown, including direct anterior, anteromedial, and anterolateral approaches. These variants may be selected based on prior scars, soft-tissue quality, vascularity, and the need for adjunctive procedures. TAA = total ankle arthroplasty.

The standard anterior approach uses an incision over the front of the ankle, typically between the tibialis anterior tendon and the extensor hallucis longus, providing a direct corridor to the tibiotalar joint (Fig. 1-A). Variations include direct anterior, anteromedial, and anterolateral incisions, with selection guided primarily by prior scars, soft-tissue quality, vascularity, and the need for adjunctive procedures rather than definitive comparative trials (Fig. 2)^23-25^. The anteromedial approach, positioned just medial to the tibialis anterior tendon, lies between the anterior tibial and posterior tibial angiosomes and may reduce wound complications in higher-risk patients^23,24^. The anterolateral approach, located lateral to the extensor hallucis longus, can improve access to the lateral gutter and talar dome but may increase risk to branches of the superficial peroneal nerve, particularly with distal extension^25,26^. The anterior capsule is longitudinally incised, and osteophyte excision is often performed to improve visualization, although some patient-specific guide systems preserve these osteophytes to facilitate guide placement. This exposure generally allows precise bone preparation without osteotomy, but severe deformity or dense anterior scarring may limit access^27^. Despite these nuances, it remains the most widely used approach, provided incision placement accounts for soft-tissue quality and vascularity^28^.

### Lateral Transfibular Approach

The lateral transfibular approach provides extensile access through a lateral incision centered over the fibula. A distal fibular osteotomy opens the lateral column and exposes the tibiotalar and subtalar joints (Fig. 1-B)^28,29^. This corridor provides broad visualization for deformity correction and implant alignment, particularly in ankles with significant varus or valgus malalignment^28,30^. In addition, it helps restore the anatomic contour of the tibia and talus while limiting bone resection^31^. By preserving the anterior skin and angiosome, this approach may reduce wound complications compared with anterior-based incisions^21,30^. Drawbacks include fibular fixation, nonunion risk, hardware irritation, and limited medial gutter visualization, sometimes requiring fluoroscopic assistance^21,29^.

Fibular osteotomy fixation may be performed with plates, screws, or intramedullary nails depending on osteotomy configuration and implant-specific technique. Long oblique fibular osteotomy has been associated with less wound dehiscence and fewer hardware removals than short oblique osteotomy, whereas intramedullary fixation may reduce hardware-related reoperation at the cost of higher reported nonunion rates^28,32^. Overall, the lateral transfibular approach provides broad exposure and favorable soft-tissue outcomes but adds bony morbidity and technical complexity compared with anterior approaches.

## Soft Tissue Perfusion and Wound Complications

**Fig. 3:**
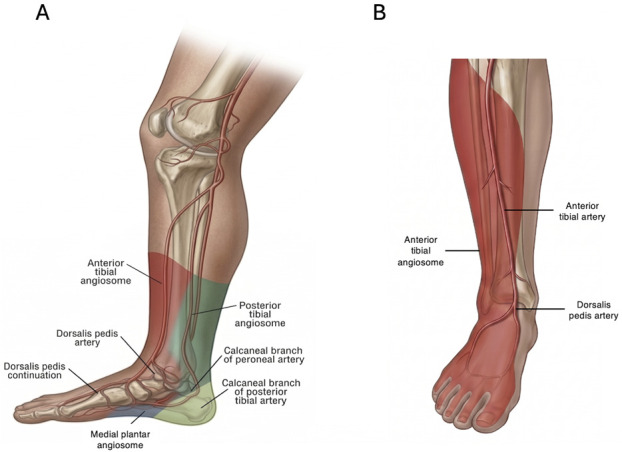
Angiosome territories relevant to TAA planning. **Fig. 3-A** A medial-oblique view of the distal leg, ankle, and foot. The red territory represents the anterior tibial angiosome with dorsalis pedis continuation, the dark green territory represents the posterior tibial angiosome, the blue territory represents the medial plantar angiosome, and the light green territory represents the calcaneal branch of the posterior tibial artery. **Fig. 3-B** An anterior view of the distal leg, ankle, and foot, highlighting the anterior tibial angiosome and the course of the anterior tibial artery as it continues distally as the dorsalis pedis artery. This schematic illustrates the vascular anatomy most relevant to anterior incision planning and soft-tissue preservation during TAA. TAA = total ankle arthroplasty.

The ankle’s thin skin, limited subcutaneous tissue, and distinct vascular territories create a narrow margin for safe exposure. Incision placement relative to the angiosomes supplied by the anterior tibial, posterior tibial, and peroneal arteries directly affects wound-healing potential (Fig. 3)^33,34^. Disruption of these vascular zones can lead to flap necrosis, infection, or hardware exposure, threatening both implant and limb survival.

Anterior-based incisions carry the greatest wound complication risk because they traverse thin, poorly perfused skin, and cross multiple angiosomes^34^. Reported wound complication rates generally range from 10% to 20% and may exceed 30% in high-risk patients^35-37^. Full-thickness anterior wound necrosis is a well-recognized and potentially devastating complication that may require flap coverage^38^. Risk reduction depends on meticulous soft-tissue handling, limited tourniquet time, postoperative splinting or casting, and selective use of incisional negative-pressure therapy^23,39,40^. Anteromedial incision placement may improve perfusion and reduce wound-complication rates to 3% to 4% in higher-risk cohorts^24^. A posteromedial accessory incision has been described to facilitate posterior capsular release and protected retractor placement without increasing technique-related complications^33,41^.

Lateral incisions generally preserve better prefusion by avoiding the thin anterior skin and remaining within more robust vascular territories. The lateral transfibular approach maintains blood flow around the lateral malleolus and minimizes dorsal angiosome compromise^21^. Clinical series report low wound-complication and infection rates, with fibular irritation or delayed osteotomy union more common than incisional breakdown^30^.

## Technical Considerations and Learning Curve

Technical success in TAA depends on exposure, instrumentation, implant compatibility, and surgeon familiarity with the chosen approach^42^.

### Instrumentation and Implant Specifics

Approach dictates implant compatibility because TAA instrumentation is engineered for a specific direction of insertion. The anterior approach is compatible with most modern fixed-bearing and mobile-bearing implants because most systems were designed for anterior access^8^. Patient-specific instrumentation is now available for several anterior TAA platforms, and modern anterior systems incorporate stems, porous coatings, and highly cross-linked polyethylene, narrowing some of the historical differences between anterior and lateral implants^43-45^. By contrast, the lateral transfibular approach requires side-entry systems such as the Zimmer/Biomet *Trabecular Metal Total Ankle* (Zimmer/Biomet, Warsaw, IN), whose guides mount laterally and insert through the fibular window^30^.

### Surgeon Experience and Learning Curve

TAA has a notable learning curve, and early surgical experience influences complication rates and alignment accuracy. Complication rates decline substantially after approximately 20 to 30 cases^21,46^. Higher surgeon volume is associated with improved inpatient outcomes; surgeons performing ≥21 TAAs per year had significantly lower complication rates, shorter hospital stays, and reduced costs compared with lower-volume surgeons^47^. The anterior approach is generally more intuitive for surgeons familiar with anterior ankle surgery, whereas the lateral transfibular approach requires additional familiarity with fibular osteotomy, fixation, and use of the external alignment jig^21^. Although the jig is a powerful positioning tool, it may be challenging to navigate early in a surgeon’s experience.

## Functional and Radiographic Outcomes

### Functional Recovery and Patient-Reported Outcomes

Functional recovery after TAA is determined primarily by implant design, surgical accuracy, and patient-specific factors, while surgical approach exerts a secondary but measurable effect on early recovery^48,49^. Longitudinal studies show pain and function improvements are sustained over time and not primarily dependent on approach^50,51^. Contemporary reviews report significant patient-reported outcome measure (PROM) improvement after TAA, with satisfaction rates typically exceeding 90%^31^.

Postoperative protocols differ modestly between approaches. The anterior approach often delays weight-bearing until incision healing is confirmed because of greater wound risk^36^. The lateral transfibular approach typically restricts weight-bearing for approximately 6 weeks to allow fibular osteotomy union^28,29^. These differences may affect early rehabilitation, with lateral-approach patients experiencing temporary fibular discomfort and anterior-approach patients reporting incision tightness or delayed wound healing^28,35^. A randomized trial found that early mobilization yields outcomes comparable with 6 weeks of immobilization, regardless of approach, and early recovery differences generally resolve by 2 to 3 months^51,52^.

Across modern lateral-approach series, American Orthopaedic Foot & Ankle Society Ankle-Hindfoot Scores improved from approximately 32 to 34 preoperatively to 80 to 88 postoperatively, and Visual Analogue Scale pain scores decreased from 7.8 to 8.5 to 1.3 to 2.3^30,53,54^. However, American Orthopaedic Foot & Ankle Society scores should be interpreted cautiously, as this instrument has recognized limitations and has not been validated as a PROM. SF-12 Physical and Mental Component Scores also improved substantially, from approximately 30 to 45-47 and 43 to 52-56, respectively^28,50^. Five-year data from lateral trabecular metal systems showed Foot and Ankle Outcome Score improvements exceeding minimal clinically important differences across pain, symptoms, function, sports, and quality of life, with gains plateauing after 1 year and maintained through midterm follow-up^55^. Patients with lower preoperative functional scores often demonstrate greater gains, with average total motion arcs of 34 to 40° and roughly 20% increased participation in low-impact sports after TAA^31^.

Range-of-motion improvements are consistent across approaches, with dorsiflexion increasing from 6 to 7 to 13 to 24° and plantarflexion from 9 to 19 to 16 to 29°^20,28,30^. Gait analyses confirm that TAA restores near-normal ankle kinematics and outperforms arthrodesis, with more than 84% of patients reporting satisfaction following either anterior or lateral TAA^56-58^.

### Radiographic and Biomechanical Outcomes

Both anterior and lateral approaches can achieve accurate alignment and stable radiographic results with modern instrumentation and planning. The lateral transfibular approach offers a coronal-plane correction advantage through direct realignment with fibular osteotomy^30^. Contemporary lateral-approach series show neutral alignment, minimal radiolucency, and no talar component subsidence, whereas anterior-approach cohorts achieve similarly stable results with adjunctive procedures as needed^30,53^. When present, radiolucent lines are generally nonprogressive and asymptomatic^46^. Both approaches improve biomechanics and load distribution, reducing adjacent-joint stress and demonstrate comparable radiographic durability^21,58^.

### Implant Survivorship and Comparative Evidence

Implant survivorship remains high across all approaches, but comparisons between anterior and lateral TAA should be interpreted cautiously because differences may reflect implant-specific design features, including fixation method and bearing type, rather than surgical approach alone^21,49^. Lateral-approach studies report 92% to 100% survivorship at 2 to 6 years, with complication rates of 10% to 36%, largely reflecting wound issues or fibular hardware irritation rather than implant failure^30,54,59^. In a long-term study of 130 lateral-approach cases, no implant loosening, subsidence, or revisions were observed through more than 5 years of follow-up^30^. Five-year data from the lateral trabecular metal system reported 92% survivorship, a 23% reoperation rate, and PROM improvement, with most secondary procedures limited to medial gutter debridement or fibular plate removal^55^. Registry and prospective data further support strong survivorship of laterally inserted trabecular metal systems, including 95% survivorship at 3 years in 239 Swedish registry cases, a 1.8% revision rate at 5 years for 420 prostheses in Australian registry data, and 100% versus 94.88% survivorship in cemented and cementless cohorts at mean follow-up of 7.2 and 6.2 years, respectively^60-62^.

Anterior fixed-bearing systems also demonstrate strong survivorship. Prospective multicenter data for the Infinity system showed a 1% revision rate in early follow-up, with later series reporting 91.1% survivorship at 8 years, while the UK National Joint Registry reported 90.2% survivorship at 5 years overall^44,63-65^. **INBONE II (Wright Medical Technology, Arlington, Tennessee)** has similarly demonstrated 98% survivorship at 5 years and 93.2% survivorship at minimum 10 years^44,66^. However, anteriorly based TAA systems remain heterogenous, with variable follow-up that limits direct comparison across studies.

## Approach Selection and Clinical Decision Framework

Selecting the optimal surgical approach for TAA requires balancing patient factors, technical considerations, and surgeon experience. The decision should be individualized to maximize exposure, preserve soft-tissue perfusion, and achieve alignment while minimizing wound and neurovascular complications^24,30^. In patients with compromised soft-tissue envelopes or vascular disease, collaboration with plastic or vascular surgery may help optimize incision planning and wound healing^67^. TAA is generally favored when motion preservation is desired and adequate bone stock, alignment, and soft-tissue conditions permit reconstruction, whereas arthrodesis may be safer in the setting of severe deformity not amenable to correction, unreconstructable soft-tissue compromise, active infection, or profound neuropathy^4,30,34^_._

### Patient-Specific Factors

Soft-tissue quality, deformity magnitude and plane, and prior surgery or hardware history are key determinants of approach selection. The anterior approach should be avoided in patients with compromised anterior skin, including scarring, prior incisions, or poor vascularity^34,35^. If anterior skin is compromised but lateral tissue is intact, a lateral transfibular approach may be preferred because it preserves perfusion and avoids poorly vascularized areas^50^. Conversely, when lateral skin is scarred or grafted, an anterior or anteromedial incision is favored. When both zones are compromised, staged reconstruction or arthrodesis may be safer. Incisions should respect angiosome boundaries and avoid watershed zones whenever possible^34,68^.

Neuropathic arthropathy or clinically significant loss of protective sensation should be considered a contraindication or strong relative contraindication to TAA. Impaired sensation, diminished muscular control, and repetitive unrecognized loading increase the risks of instability, loosening, wound complications, and early failure. In these patients, arthrodesis is often the safer reconstructive option^69,70^.

For ankles with coronal deformity exceeding approximately 20°, the lateral transfibular approach offers superior visualization and correction by allowing release of the tight side and realignment through fibular osteotomy^30^. The anterior approach can manage mild to moderate deformities of approximately 5° to 15° when combined with targeted soft-tissue releases and osteotomies^71^. Sagittal contractures, such as equinus, may be addressed with Achilles or gastrocnemius lengthening regardless of approach^72,73^. For rotational malalignment or subluxation, the lateral corridor may provide a mechanical advantage via direct manipulation of the fibula and talus^74^.

Lateral access is advantageous when anterior hardware or scarring complicates reentry, whereas a well-healed anterior incision without implant interference supports reuse^33^. Overlapping or crossing old scars should be avoided, and existing hardware should be assessed for retention, removal, or reuse during planning. When prior scars or compromised skin limit a standard direct anterior incision, an anteromedial or anterolateral variant may be preferable, and accessory or separate incisions may help avoid crossing tenuous soft-tissue zones^24,25,75^.

### Operative and Technical Considerations

Concomitant procedures should be integrated into preoperative planning. The lateral transfibular approach is appropriate for subtalar fusion, calcaneal osteotomy, selected tendon transfers, and lateral soft-tissue balancing procedures through same exposure or adjunctive incisions, depending on pathology and implant-specific workflow^50^. A practical limitation of lateral TAA systems is constrained revision flexibility, as failed lateral implants often require revision through an anterior approach because a dedicated revision system for the lateral platform was not developed^76^. Separate medial or posteromedial accessory incisions may complement either approach when medial balancing, posterior release, or adjunctive procedures cannot be addressed safely through the primary window^33,41,75^. Separate incisions have been used for medial malleolar fixation, lateral ligament repair, and first metatarsal osteotomy without increased short-term complications^75^. When additional exposure is required, the anterior approach may be extended proximally for tibial work or distally for selected midfoot procedures, and concomitant tibial or fibular osteotomy can be performed with high union rates^77^. Tourniquet use should be judicious, as prolonged duration and excessive pressure may increase wound complications and postoperative pain; individualized pressure settings and staged procedures may be considered when multiple reconstructions are required^39,67^.

## Conclusion

TAA is a dependable motion-preserving treatment for end-stage ankle arthritis. Anterior and lateral approaches can provide predictable outcomes when appropriately matched to patient anatomy, deformity, soft-tissue considerations, implant requirements, and surgeon expertise. Ultimately, approach selection should be individualized and strategy-driven, as successful outcomes depend less on the chosen corridor than on patient selection, meticulous technique, soft-tissue management, and alignment accuracy. Standardized reporting and approach-stratified outcome data will be important to refine evidence-based guidance for TAA approach selection.

## References

[1] Herrera-Pérez M González-Martín D Vallejo-Márquez M Godoy-Santos AL Valderrabano V Tejero S. Ankle osteoarthritis aetiology. J Clin Med. 2021;10(19):4489. 34640504 10.3390/jcm10194489 PMC8509242

[2] Anastasio AT Lau B Adams S. Ankle osteoarthritis. J Am Acad Orthop Surg. 2024;32(16):738-46. 38810230 10.5435/JAAOS-D-23-00743

[3] Valderrabano V Horisberger M Russell I Dougall H Hintermann B. Etiology of ankle osteoarthritis. Clin Orthopaedics Relat Res. 2009;467(7):1800-6. 10.1007/s11999-008-0543-6 PMC2690733 18830791

[4] Teehan E Demetracopoulos C. Outcomes of total ankle replacement. Orthop Clin North America. 2024;55(4):503-12. 10.1016/j.ocl.2024.05.002 39216955

[5] Goldberg AJ Chowdhury K Bordea E Blackstone J Brooking D Deane EL Hauptmannova I Cooke P Cumbers M Skene SS Doré CJ. Total ankle replacement versus ankle arthrodesis for patients aged 50-85 years with end-stage ankle osteoarthritis: the TARVA RCT. Health Technol Assess. 2023;27(05):1-80. 10.3310/PTYJ1146 PMC10150410 37022932

[6] Fanelli D Mercurio M Castioni D Sanzo V Gasparini G Galasso O. End-stage ankle osteoarthritis: arthroplasty offers better quality of life than arthrodesis with similar complication and re-operation rates-an updated meta-analysis of comparative studies. Int Orthopaedics. 2021;45(9):2177-91. 10.1007/s00264-021-05053-x 33944980

[7] Sangeorzan BJ Ledoux WR Shofer JB Davitt J Anderson JG Bohay D Coetzee JC Maskill J Brage M Norvell DC. Comparing 4-year changes in patient-reported outcomes following ankle arthroplasty and arthrodesis. J Bone Joint Surg. 2021;103(10):869-78. 33983146 10.2106/JBJS.20.01357 PMC11807391

[8] Henricson A Nilsson JÅ Carlsson A. 10-year survival of total ankle arthroplasties: a report on 780 cases from the Swedish ankle register. Acta Orthopaedica. 2011;82(6):655-9. 22066551 10.3109/17453674.2011.636678 PMC3247880

[9] Liu S Wang Y Zhang M Wei P Li Y Wang T Meng Q. A comparative study of modern total ankle replacement and ankle arthrodesis for ankle osteoarthritis at different follow-up times: a systematic review and meta-analysis. Int Orthopaedics. 2023;47(6):1493-510. 10.1007/s00264-023-05753-6 36897362

[10] Norvell DC Ledoux WR Shofer JB Hansen ST Davitt J Anderson JG Bohay D Coetzee JC Maskill J Brage M Houghton M Sangeorzan BJ. Effectiveness and safety of ankle arthrodesis versus arthroplasty: a prospective multicenter study. J Bone Joint Surg. 2019;101(16):1485-94. 31436657 10.2106/JBJS.18.01257 PMC7001770

[11] Odum SM Van Doren BA Anderson RB Davis WH. In-hospital complications following ankle arthrodesis versus ankle arthroplasty: a matched cohort study. J Bone Joint Surg. 2017;99(17):1469-75. 28872529 10.2106/JBJS.16.00944

[12] Stavrakis AI SooHoo NF. Trends in complication rates following ankle arthrodesis and total ankle replacement. J Bone Joint Surg. 2016;98(17):1453-8. 27605689 10.2106/JBJS.15.01341

[13] Karzon AL Kadakia RJ Coleman MM Bariteau JT Labib SA. The rise of total ankle arthroplasty use: a database analysis describing case volumes and incidence trends in the United States between 2009 and 2019. Foot Ankle Int. 2022;43(11):1501-10. 36050924 10.1177/10711007221119148

[14] Singh JA Ramachandran R. Time trends in total ankle arthroplasty in the USA: a study of the National Inpatient Sample. Clin Rheumatol. 2016;35(1):239-45. 24907036 10.1007/s10067-014-2703-2 PMC4258518

[15] Delco ML Kennedy JG Bonassar LJ Fortier LA. Post-traumatic osteoarthritis of the ankle: a distinct clinical entity requiring new research approaches. J Orthopaedic Res. 2017;35(3):440-53. 10.1002/jor.23462 PMC5467729 27764893

[16] Anastasio AT Kim BI Wixted CM DeOrio JK Nunley JA 2nd Easley ME Adams SB. Younger patients undergoing total ankle arthroplasty experience higher complication rates and worse functional outcomes. J Bone Joint Surg. 2024;106(1):10-20. 37922342 10.2106/JBJS.23.00122

[17] Vickerstaff JA Miles AW Cunningham JL. A brief history of total ankle replacement and a review of the current status. Med Eng Phys. 2007;29(10):1056-64. 17300976 10.1016/j.medengphy.2006.11.009

[18] Easley ME Vertullo CJ Urban CW Nunley JA. Total ankle arthroplasty. J Am Acad Orthopaedic Surgeons. 2002;10(3):157-67. 10.5435/00124635-200205000-00002 12041937

[19] Chou LB Coughlin MT Hansen S Jr Haskell A Lundeen G Saltzman CL Mann RA. Osteoarthritis of the ankle: the role of arthroplasty. J Am Acad Orthopaedic Surg. 2008;16(5):249-59. 10.5435/00124635-200805000-00003 18460685

[20] Barg A Bettin CC Burstein AH Saltzman CL Gililland J. Early clinical and radiographic outcomes of trabecular metal total ankle replacement using a transfibular approach. J Bone Joint Surg. 2018;100(6):505-15. 29557867 10.2106/JBJS.17.00018

[21] Gagné OJ Penner M Wing K Veljkovic A Younger AS. Reoperation profile of lateral vs anterior approach ankle arthroplasty. Foot Ankle Int. 2020;41(7):834-8. 32441532 10.1177/1071100720920276

[22] Waugh TR Evanski PM McMaster WC. Irvine ankle arthroplasty. Prosthetic design and surgical technique. Clin Orthop Relat Res. 1976;114:180-4. 1261110

[23] LaPorta GA Schnack LL Begum M Yates EA Oexeman S Rodriguez-Collazo ER. A retrospective review of soft-tissue complications in total talus replacement and total ankle replacement using the orthoplastic anteromedial approach. J Am Podiatric Med Assoc. 2025;115(4):23-090. 10.7547/23-090 40875457

[24] Halai MM Pinsker E Daniels TR. Effect of novel anteromedial approach on wound complications following ankle arthroplasty. Foot Ankle Int. 2020;41(10):1198-205. 32683898 10.1177/1071100720937247

[25] Hirao M Ebina K Etani Y Kaneshiro S Tsuboi H Noguchi T Okamura G Kunugiza Y Nakaya H Nishikawa M Tsuji S Takahi K Owaki H Hashimoto J. Modified anterolateral approach for total ankle arthroplasty. Foot Ankle Orthop. 2021;6(2):24730114211013342. 35097449 10.1177/24730114211013342 PMC8725997

[26] McAlister JE DeMill SL Hyer CF Berlet GC. Anterior approach total ankle arthroplasty: superficial peroneal nerve branches at risk. J Foot Ankle Surg. 2016;55(3):476-9. 26884263 10.1053/j.jfas.2015.12.013

[27] Clough T Bodo K Majeed H Davenport J Karski M. Survivorship and long-term outcome of a consecutive series of 200 Scandinavian Total Ankle Replacement (STAR) implants. Bone Joint J. 2019;101-B(1):47-54. 30601052 10.1302/0301-620X.101B1.BJJ-2018-0801.R1

[28] Usuelli FG Indino C Maccario C Manzi L Romano F Aiyer A Kaplan JRM. A modification of the fibular osteotomy for total ankle replacement through the lateral transfibular approach. J Bone Joint Surg. 2019;101(22):2026-35. 31764365 10.2106/JBJS.19.00307

[29] DeVries JG Derksen TA Scharer BM Limoni R. Perioperative complications and initial alignment of lateral approach total ankle arthroplasty. J Foot Ankle Surg. 2017;56(5):996-1000. 28645548 10.1053/j.jfas.2017.04.016

[30] Day J Fletcher AN Motsay M Manchester M Arthur M Zhang Z Schon LC. Outcomes of transfibular total ankle arthroplasty: clinical and radiographic analysis of 130 cases with minimum 5-year follow-up. J Bone Joint Surg. 2025;107(12):e61. 40299950 10.2106/JBJS.24.00983

[31] Shaffrey I Henry J Demetracopoulos C. An evaluation of the total ankle replacement in the modern era: a narrative review. Ann Transl Med. 2024;12(4):71. 39118953 10.21037/atm-23-1569 PMC11304414

[32] Schafer KA Day J Min BK Motsay M Zhang Z Schon LC. Fibular osteotomy healing in transfibular total ankle arthroplasty. Foot Ankle Int. 2026;47(3):318-23. 41439394 10.1177/10711007251401489

[33] Tejero S Chans-Veres J Prada-Chamorro E DeOrio JK. Protective approach for anatomical structures at risk in total ankle replacement. J Foot Ankle Surg. 2021;60(2):417-20. 33358384 10.1053/j.jfas.2020.06.002

[34] Attinger CE Evans KK Bulan E Blume P Cooper P. Angiosomes of the foot and ankle and clinical implications for limb salvage: reconstruction, incisions, and revascularization. Plast Reconstr Surg. 2006;117(7 suppl):261S-93S. 16799395 10.1097/01.prs.0000222582.84385.54

[35] Reb CW Watson BC Fidler CM Van Dyke B Hyer CF Berlet GC Prissel MA. Anterior ankle incision wound complications between total ankle replacement and ankle arthrodesis: a matched cohort study. J Foot Ankle Surg. 2021;60(1):47-50. 33168440 10.1053/j.jfas.2020.04.015

[36] Smeeing DPJ Briet JP van Kessel CS Segers MM Verleisdonk EJ Leenen LPH Houwert RM Hietbrink F. Factors associated with wound- and implant-related complications after surgical treatment of ankle fractures. J Foot Ankle Surg. 2018;57(5):942-7. 30005967 10.1053/j.jfas.2018.03.050

[37] Lanzetti RM Lupariello D Venditto T Guzzini M Ponzo A De Carli A Ferretti A. The role of diabetes mellitus and BMI in the surgical treatment of ankle fractures. Diabetes Metab Res Rev. 2018;34(2):e2954. 10.1002/dmrr.2954 29031012

[38] Hulsker CC Kleinveld S Zonnenberg CB Hogervorst M van den Bekerom MP. Evidence-based treatment of open ankle fractures. Arch Orthop Trauma Surg. 2011;131(11):1545-53. 21713539 10.1007/s00402-011-1349-7 PMC3228491

[39] Cifaldi A McGlone W McKee T Bonvillian J Blacklidge D Miller JM Elliott B. Anterior ankle incision healing complications with and without tourniquet use: a retrospective comparative cohort study. J Foot Ankle Surg. 2023;62(2):304-9. 36127242 10.1053/j.jfas.2022.08.006

[40] Matsumoto T Parekh SG. Use of negative pressure wound therapy on closed surgical incision after total ankle arthroplasty. Foot Ankle Int. 2015;36(7):787-94. 25736324 10.1177/1071100715574934

[41] Stoops TK Sanders RW. Posteromedial accessory incision for posterior capsular release and retractor placement in a total ankle replacement. Foot Ankle Int. 2022;43(5):733-7. 35135339 10.1177/10711007211071142

[42] Tennant JN Rungprai C Pizzimenti MA Goetz J Phisitkul P Femino J Amendola A. Risks to the blood supply of the talus with four methods of total ankle arthroplasty: a cadaveric injection study. J Bone Joint Surg. 2014;96(5):395-402. 24599201 10.2106/JBJS.M.01008

[43] Tham A Rubin J Bieganowski T Butler JJ Konar K Walls RJ Schon LC Kennedy JG. Patient-specific vs standard instrumentation in total ankle arthroplasty: a systematic review and meta-analysis of short-term outcomes. Foot Ankle Int. 2026;47(3):304-17. 41589398 10.1177/10711007251405239

[44] Gagne OJ Day J Kim J Caolo K O'Malley MJ Deland JT Ellis SJ Demetracopoulos CA. Midterm survivorship of the INBONE II total ankle arthroplasty. Foot Ankle Int. 2022;43(5):628-36. 34905959 10.1177/10711007211060047

[45] Schipper ON Haddad SL Fullam S Pourzal R Wimmer MA. Wear characteristics of conventional ultrahigh-molecular-weight polyethylene versus highly cross-linked polyethylene in total ankle arthroplasty. Foot Ankle Int. 2018;39(11):1335-44. 30019605 10.1177/1071100718786501

[46] Arshad Z Haq II Bhatia M. Learning curve of total ankle arthroplasty: a systematic review. Arch Orthop Trauma Surg. 2023;144(2):591-600. 37917408 10.1007/s00402-023-05064-w

[47] Basques BA Bitterman A Campbell KJ Haughom BD Lin J Lee S. Influence of surgeon volume on inpatient complications, cost, and length of stay following total ankle arthroplasty. Foot Ankle Int. 2016;37(10):1046-51. 27540010 10.1177/1071100716664871

[48] Cunningham DJ DeOrio JK Nunley JA Easley ME Adams SB. The effect of patient characteristics on 1 to 2-year and minimum 5-year outcomes after total ankle arthroplasty. J Bone Joint Surg. 2019;101(3):199-208. 30730479 10.2106/JBJS.18.00313

[49] Lefrancois T Younger A Wing K Penner MJ Dryden P Wong H Daniels T Glazebrook M. A prospective study of four total ankle arthroplasty implants by non-designer investigators. J Bone Joint Surg. 2017;99(4):342-8. 28196036 10.2106/JBJS.16.00097

[50] Mosca M Caravelli S Vocale E Maitan N Grassi A Massimi S Fuiano M Zaffagnini S. Clinical-radiological outcomes and complications after total ankle replacement through a lateral transfibular approach: a retrospective evaluation at a mid-term follow-up. Int Orthop. 2021;45(2):437-43. 32666242 10.1007/s00264-020-04709-4

[51] Hendy BA McDonald EL Nicholson K Rogero R Shakked R Pedowitz DI Raikin SM. Improvement of outcomes during the first two years following total ankle arthroplasty. J Bone Joint Surg. 2018;100(17):1473-81. 30180055 10.2106/JBJS.17.01021

[52] Ramaskandhan J Kakwani R Kometa S Hewart P Rawlings D Chockalingam N Siddique M. Randomized controlled trial comparing early mobilization vs six weeks of immobilization in a walking cast following total ankle replacement. J Foot Ankle Surg. 2023;62(4):595-600. 36710141 10.1053/j.jfas.2022.12.005

[53] Maccario C Paoli T Romano F D'Ambrosi R Indino C Usuelli FG. Transfibular total ankle arthroplasty: a new reliable procedure at five-year follow-up. Bone Joint J. 2022;104-B(4):472-8. 35360940 10.1302/0301-620X.104B4.BJJ-2021-0167.R5

[54] Abarquero-Diezhandino A Vacas Sánchez E Diaz Fernandez R Vilá y Rico J Rico J. Results of transfibular total ankle arthroplasty. a series of 50 implants. J Foot Ankle Surg. 2023;62(4):671-5. 36941143 10.1053/j.jfas.2023.02.005

[55] Kim J Gagne OJ Rajan L Caolo K Sofka C Ellis SJ Demetracopoulos CA Deland JT. Clinical outcomes of the lateral trabecular metal total ankle replacement at a 5-year minimum follow-up. Foot Ankle Spec. 2023;16(3):288-99. 36482702 10.1177/19386400221139525

[56] Oliver SM Coetzee JC Nilsson LJ Samuelson KM Stone RM Fritz JE Giveans MR. Early patient satisfaction results on a modern generation fixed-bearing total ankle arthroplasty. Foot Ankle Int. 2016;37(9):938-43. 27162222 10.1177/1071100716648736

[57] Younger AS Wing KJ Glazebrook M Daniels TR Dryden PJ Lalonde KA Wong H Qian H Penner M. Patient expectation and satisfaction as measures of operative outcome in end-stage ankle arthritis: a prospective cohort study of total ankle replacement versus ankle fusion. Foot Ankle Int. 2015;36(2):123-34. 25645533 10.1177/1071100714565902

[58] Singer S Klejman S Pinsker E Houck J Daniels T. Ankle arthroplasty and ankle arthrodesis: gait analysis compared with normal controls. J Bone Joint Surg Am. 2013;95(24):e191(1-10). 24352777 10.2106/JBJS.L.00465

[59] Fletcher AN Day J Motsay M Manchester M Zhang Z Schon LC. Transfibular total ankle arthroplasty: clinical, functional, and radiographic outcomes and complications at a minimum of 5-year follow-up. Foot Ankle Int. 2025;46(1):1-8. 39526761 10.1177/10711007241290222

[60] Henricson A Undén A Carlsson Å Jehpsson L Rosengren B. Outcomes of trabecular metal total ankle replacement: a longitudinal observational cohort study of 239 consecutive cases from the Swedish Ankle Registry. Acta Orthop. 2022;93:689-95. 35919022 10.2340/17453674.2022.4387 PMC9348131

[61] Stavrou P Lewis PL McAuliffe MJ . Demographics and outcome of ankle arthroplasty: Supplementary report. In: Hip, Knee & Shoulder Arthroplasty. Australian Orthopaedic Association National Joint Replacement Registry, AOA, Adelaide; 2024.

[62] Younger ASE Cholewa J Ellington JK Giza E Patsalis T Krause F Usuelli FG. Outcomes of laterally inserted trabecular metal total ankle arthroplasty comparing cemented and cementless rail fixation: a prospective, longitudinal study. Foot Ankle Int. 2026;47(1):22-33. 41410141 10.1177/10711007251386032

[63] Townshend DN Bing AJF Clough TM Sharpe IT Goldberg A., UK INFINITY Study Group. Early experience and patient-reported outcomes of 503 INFINITY total ankle arthroplasties. Bone Joint J. 2021;103-B(7):1270-6. 34192928 10.1302/0301-620X.103B7.BJJ-2020-2058.R2 PMC9948432

[64] Dahill M Kostusiak M Dean M Hughes A Kakwani R Murty A Townshend D Sharpe I. Midterm survivorship of 106 infinity total ankle replacements: a case series from 2 non-designer UK centers. Foot Ankle Int. 2025;46(8):855-61. 40568754 10.1177/10711007251341313

[65] Jennison T Ukoumunne OC Lamb S Sharpe I Goldberg A. Risk factors for failure of total ankle replacements: a data linkage study using the national joint registry and NHS digital. Foot Ankle Int. 2023;44(7):596-603. 37345846 10.1177/10711007231176512

[66] Campbell M Chandler C Burger J Hodgens B Teachey W Hietpas K Anderson RB Cohen B Jones CP Ford S. Long-term survivorship of INBONE II total ankle arthroplasty. Foot Ankle Int. 2026:10711007251412377. 10.1177/10711007251412377 41677768

[67] Gross CE Garcia R Adams SB DeOrio JK Easley ME Nunley JA 2nd. Soft tissue reconstruction after total ankle arthroplasty. Foot Ankle Int. 2016;37(5):522-7. 26686238 10.1177/1071100715624206

[68] Day J Principe PS Caolo KC Fragomen AT Rozbruch SR Ellis SJ. A staged approach to combined extra-articular limb deformity correction and total ankle arthroplasty for end-stage ankle arthritis. Foot Ankle Int. 2021;42(3):257-67. 33185124 10.1177/1071100720965120

[69] Deorio JK Easley ME. Total ankle arthroplasty. Instr Course Lect. 2008;57:383-413. 18399599

[70] Dujela MD Berlet GC Houng BE Hyer CF. Comparison of dynamic versus static locked retrograde tibiotalocalcaneal arthrodesis with intramedullary nail fixation: evaluation of the RAIN database. J Foot Ankle Surg. 2023;62(4):657-60. 36941141 10.1053/j.jfas.2023.02.002

[71] Lee GW Lee KB. Outcomes of total ankle arthroplasty in ankles with >20° of coronal plane deformity. J Bone Joint Surg. 2019;101(24):2203-11. 31596804 10.2106/JBJS.19.00416

[72] Firth GB McMullan M Chin T Ma F Selber P Eizenberg N Wolfe R Graham HK. Lengthening of the gastrocnemius-soleus complex: an anatomical and biomechanical study in human cadavers. J Bone Joint Surg. 2013;95(16):1489-96. 23965699 10.2106/JBJS.K.01638

[73] Tagoe MT Reeves ND Bowling FL. Is there still a place for achilles tendon lengthening? Diabetes Metab Res Rev. 2016;32(S1):227-31. 26452341 10.1002/dmrr.2745

[74] Park JJ Son WS Woo IH Park CH. Combined transfibular and anterior approaches increase union rate and decrease non-weight-bearing periods in ankle arthrodesis: combined approaches in ankle arthrodesis. J Clin Med. 2021;10(24):5915. 34945209 10.3390/jcm10245915 PMC8706894

[75] Criswell B Hunt K Kim T Chou L Haskell A. Association of short-term complications with procedures through separate incisions during total ankle replacement. Foot Ankle Int. 2016;37(10):1060-4. 27283155 10.1177/1071100716651964

[76] Williams JR Wegner NJ Sangeorzan BJ Brage ME. Intraoperative and perioperative complications during revision arthroplasty for salvage of a failed total ankle arthroplasty. Foot Ankle Int. 2015;36(2):135-42. 25288333 10.1177/1071100714554452

[77] Adams SB Steele JR Demetracopoulos CA Nunley JA Easley ME DeOrio JK. Results of tibia and fibula osteotomies performed concomitant to total ankle replacement. Foot Ankle Int. 2020;41(3):259-66. 32134716 10.1177/1071100719892221

